# Environmental Lead after Hurricane Katrina: Implications for Future Populations

**DOI:** 10.1289/ehp.1103774

**Published:** 2011-11-03

**Authors:** Felicia A. Rabito, Shahed Iqbal, Sara Perry, Whitney Arroyave, Janet C. Rice

**Affiliations:** 1 Department of Epidemiology, and; 2 Department of Biostatistics and Bioinformatics, Tulane University School of Public Health and Tropical Medicine, New Orleans, Louisiana, USA

**Keywords:** children’s health, environmental exposures, housing, lead exposure, soil pollutants

## Abstract

Background: As a result of Hurricane Katrina, > 100,000 homes were destroyed or damaged and a significant amount of sediment was deposited throughout the city of New Orleans, Louisiana. Researchers have identified the potential for increased lead hazards from environmental lead contamination of soils.

Objectives: We assessed the distribution of residential soil and dust lead 2 years poststorm and compared soil lead before and after the storm.

Methods: We conducted a cross-sectional study in New Orleans in which households were selected by stratified random sampling. A standard residential questionnaire was administered, and lead testing was performed for both the interior and exterior of homes. Logistic regression was used to identify significant predictors of interior and exterior lead levels in excess of allowable levels.

Results: One hundred nine households were enrolled; 61% had at least one lead measurement above federal standards. Of homes with bare soil, 47% had elevated lead and 27% had levels exceeding 1,200 ppm. Housing age was associated with soil lead, and housing age and soil lead were associated with interior lead. Race, income, and ownership status were not significantly associated with either interior or exterior lead levels. The median soil lead level of 560 ppm was significantly higher than the median level of samples collected before Hurricane Katrina.

Conclusions: The high prevalence (61%) of lead above recommended levels in soil and dust samples in and around residences raises concern about potential health risks to the New Orleans population, most notably children. Steps should be taken to mitigate the risk of exposure to lead-contaminated soil and dust. Further research is needed to quantify the possible contribution of reconstruction activities to environmental lead levels.

When Hurricane Katrina flooded the city of New Orleans, Louisiana, and adjacent areas in August 2005, one of many environmental health concerns was the possibility of widespread contamination of soils and sediments. To assess the hurricane’s impact, the U.S. Environmental Protection Agency (EPA) and the Louisiana Department of Environmental Quality initiated an investigation into the floodwater sediment contamination in residential neighborhoods both before the floodwaters receded and before cleanup. Sampling results indicated that residential soils contained lead; however, the U.S. EPA found that the hurricane did not significantly affect the distribution of lead because the posthurricane geography of lead distribution resembled prehurricane distributions (U.S. EPA 2005). Another lead assessment, conducted in 2006, reported a 46% decrease in median soil lead from pre-Katrina levels (Zahran et al. 2010). Both of these studies were conducted in the immediate aftermath of Hurricane Katrina and preceded the extensive renovation effort that would be required to rebuild the city.

A 2007 report by the Agency for Toxic Substances and Disease Registry (ATSDR) highlighted the potential risk of lead exposure to families returning to New Orleans in light of the extensive amount of renovation and demolition that would be required to rebuild the city. According to the 2000 U.S. Census, > 100,000 homes in New Orleans were built before 1950, an estimated 83% of which have lead hazards (ATSDR 2007). The report concluded by stating that despite surveys indicating no increase in environmental lead levels, the actual extent of lead hazards would be determined only after soil data collected subsequent to reconstruction activities became available (ATSDR 2007). A recently published survey of schoolyard soil also suggested the need for more extensive assessment of residential lead hazards (Presley et al. 2010).

To our knowledge, there have been no environmental surveys of residential hazards in New Orleans in the aftermath of Hurricane Katrina. The primary goal of the New Orleans Home Health Hazard project was to assess the burden of numerous environmental health hazards (e.g., allergen levels, mold, lead) in the homes of returning residents. The present analysis was conducted to characterize the distribution of residential soil and dust lead levels in a representative sample of New Orleans homes after the reconstruction effort in the city had begun, to compare the soil lead distribution pre- and poststorm, and to address the potential lead hazard to residents of New Orleans after the devastation of Hurricane Katrina.

## Materials and Methods

**Figure 1:**
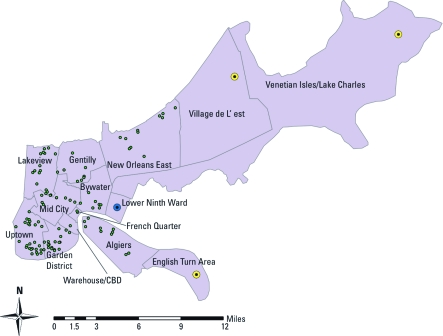
Map of New Orleans planning districts, with lead data sample locations indicated. CBD, Central Business District. Yellow dots indicate districts that were excluded from the analysis *a priori*. The blue dot indicates the district that was excluded from the analysis because of insufficient population. Green dots indicate lead sampling locations.

*Sampling and recruitment.* We conducted a cross-sectional survey of lead hazards in a representative sample of New Orleans homes. The target population was all occupied homes in New Orleans. Repopulation of the city was a dynamic process; as a result, determining population estimates was a challenge. The Louisiana Public Health Institute worked with the U.S. Census Bureau and the Centers for Disease Control and Prevention (CDC) to produce accurate and reliable estimates of the size and characteristics of the New Orleans population during the post-Hurricane Katrina recovery period (Louisiana Public Health Institute 2006). Their population estimates, which were stratified by planning district, were used for the present study. Planning districts are geographical units of the city designated by federal and state requirements for economic development planning. New Orleans is composed of 13 planning districts (Figure 1). We excluded 3 planning districts from the study because they are unique areas and do not represent the New Orleans urban core (Venetian Isles, Village de L’est, and English Turn). One other planning district, the Lower Ninth Ward, was also excluded because of the slow pace of reconstruction in that district.

Mass population shifts occurred during the period after the hurricane, making existing household sampling frames (e.g., residential rosters) inappropriate for use. Therefore, to select sample households, we used the Sewerage and Water Board address list, a roster of all residential addresses billed for water service (the best available list of reoccupied homes after the storm), as the sampling frame. Sewerage and Water Board addresses were geocoded to a census tract and assigned to the appropriate planning district.

Households were recruited from January 2007 through May 2008. Recruitment was staggered over this 17-month period to incorporate the dynamic repopulation of the city in an effort to include the maximum number of neighborhoods. Households were eligible for inclusion if the house was deemed occupied and the house was a primary residence (i.e., the location where the head of household slept ≥ 4 nights/week. Federal Emergency Management Agency (FEMA) trailers were excluded from the study. Proportionate random sampling, stratified by city planning district, was used to select 109 study households. An initial recruitment letter was hand delivered to each selected household after field staff determined that the home was occupied using a well-defined protocol developed and pilot tested by the study team. If a selected household was deemed to be ineligible, another household was randomly chosen from the relevant planning district strata. A second recruitment letter was mailed after 2 weeks if no response was received from the initial recruitment letter. Up to five recruitment letters were sent to homes deemed occupied per the study protocol. If eligible homes did not enroll after five recruitment attempts, they were deemed to be nonresponsive. Active informed consent was obtained for each enrolled household. The study was approved by the Tulane University Institutional Review Board.

*Data collection.* Each head of household was administered a survey questionnaire by a field researcher. The questionnaire was adapted from the National Survey of Lead and Allergens in Housing [Department of Housing and Urban Development (HUD) 2001]. Field staff also recorded the home’s proximity to a major road and type of house (single-family home, low-rise apartment, duplex, etc.). Lead sampling was conducted in accordance with sampling procedures as described by the U.S. EPA (1995), HUD, and the American Society for Testing and Materials (ASTM 1997a, 1997b). Surface lead dust was collected via wipe sampling from six locations in the interior of the home. Floor samples from the kitchen, bedroom, and living room floors were taken if there was bare floor, and windowsill samples were collected from the same rooms if there was a window in that room. An outdoor sample was collected from the bare soil just outside the front door of the home; if there was no bare soil in the entrance area, a sample was taken from the middle of the yard. If there was no bare soil in either of these areas, a sample was taken from the backyard. If there was no bare soil in any of these areas, no sample was taken. Results from a soil lead survey conducted between 1998 and 2000 were obtained and used to compare the distribution of lead in the city before and after Hurricane Katrina. The soil lead survey sampling protocol has been described in detail previously (Mielke et al. 2005). Briefly, soil samples were collected from 286 census tracts in New Orleans from the top 2.5 cm of bare soil following standard soil sampling procedures. Soil lead data for the present study were geocoded to a census tract. For the 55 census tracts with soil lead data from both studies, the results were compared to assess soil lead distribution pre- and poststorm.

*Sample analyses.* Dust and soil lead samples were analyzed for metals by BTS Laboratories (Waldorf, MD)—a U.S. EPA–recognized, American Industrial Hygiene Association–accredited laboratory—using U.S. EPA method 7010 with graphite furnace atomic absorption spectrophotometry (U.S. EPA 2007). The results were reported in parts per million.

*Statistical analyses.* We assessed variables known to be associated with elevated soil and dust lead levels, including age of housing, home ownership status, race, income, and proximity of the home to a busy street. For interior lead, soil lead level was also considered. Descriptive statistical analyses were performed to examine the distribution of sociodemographic and household characteristics and lead levels. Twenty-nine missing income data points were imputed from median income level of the census tract block to which the address belonged, according to the 2000 Census (U.S. Census Bureau 2003). We used logistic regression models to estimate crude and adjusted odds ratios (ORs) with 95% confidence intervals (CIs) to identify factors related to elevated soil lead levels and interior lead levels, respectively. Race, income, home ownership status, and housing located near a busy street were included in the multivariable model based on *a priori* consideration, as these variables have been shown in the literature to be strongly associated with lead levels. Wilcoxon signed-rank test for paired data was run on median census tract soil lead level to explore changes in the distribution of soil lead pre- and posthurricane. Homes that lacked sample data because there was no bare floor to sample, no windowsill in the designated room, or no bare soil around the property were excluded from the relevant analysis. All analyses were performed using SAS software (version 9.1.3; SAS Institute Inc., Cary, NC).

## Results

The sample consisted of 109 homes from nine planning districts in New Orleans; thus, power estimates were calculated on the sample size of 109 households. With an exposure prevalence rate of 61.2%, an α of 0.05, and an alternative proportion of 0.45, study power was 92%. Response rates varied from 14.2% in the French Quarter (mainly a commercial area) to 75.6% in Lakeview. The overall response rate was 32.5%. Most respondents were Caucasian (61.5%) and had an annual household income > $30,000 (67%) (Table 1). Most of the homes were built before 1946 (64.2%), and most participants were homeowners (68.8%). Four participant households (3.7%) reported receiving some form of government assistance. Nine homes (8.3%) were located near a busy street or intersection.

**Table 1:** Demographic and household characteristics of the study population (*n* = 109).

Characteristic	*n* (%)
Race/ethnicity	
African American	41 (37.6)
Caucasian	67 (61.5)
Other	1 (0.92)
Annual household income ($/year)*a*	
< 30,000	36 (33.0)
≥ 30,000	73 (67.0)
House built before 1946	70 (64.2)
≥ 1 child < 18 years of age in the home	53 (48.6)
Home ownership status	
Own	75 (68.8)
Rent	34 (31.2)
Household receives government assistance	4 (3.7)
House located near busy street	9 (8.3)
Type of house	
Single-family home	79 (72.5)
Multifamily home	30 (19.2)
**a**Imputed based on median census block income for 29 homes with missing income data.	

Using standard HUD/U.S. EPA cut points of > 40 µg/ft^2^ for floor dust and > 250 µg/ft^2^ for windowsill dust (U.S. EPA 2008a), 50.5% of homes had at least one interior sample that was elevated (Table 2). Nearly half (46.7%) of the 90 homes with bare soil had levels > 400 ppm, and 26.7% had levels > 1,200 ppm. Considering both interior and exterior samples, 61.4% of homes had at least one measurement in excess of the HUD/U.S. EPA standard.

**Table 2:** Results of interior dust lead and soil lead sampling (*n* = 109).

Sampling area*a*	HUD/U.S. EPA standard	Homes with elevated levels [*n* (%)]
Floor sample		> 40 µg/ft^2^		
Kitchen (*n* = 109)				13 (11.9)
Bedroom (*n* = 71)				9 (12.7)
Living room (*n* = 104)				15 (14.4)
Windowsill sample		> 250 µg/ft^2^		
Kitchen (*n* = 101)				24 (23.8)
Bedroom (*n* = 102)				30 (20.9)
Living room (*n* = 108)				28 (25.9)
Soil sample (*n* = 90)		> 400 ppm		42 (46.7)
		> 1,200 ppm		24 (26.7)
Elevated interior sample				55 (50.5)
Any elevated sample				67 (61.4)
**a**Floor samples were not collected from rooms without bare floors; windowsill samples were not collected from rooms without windows; and soil samples were collected from homes with bare soil near the home only.				

In unadjusted models, age of housing was the only factor significantly associated (*p* < 0.05) with elevated soil lead (OR = 82.0; 95% CI: 10.3, 651.5), whereas both age of housing (OR = 12.8; 95% CI: 4.7, 35.1) and elevated soil lead (OR = 21.2; 95% CI: 7.2, 62.7) were significantly associated with increased interior lead. In adjusted models, after controlling for housing age, soil lead remained significantly associated with interior lead levels above the HUD/U.S. EPA health-based standards (Table 3).

**Table 3:** ORs (95% CIs) for associations between sociodemographic variables and elevated interior dust lead (> 40 µg/ft^2 ^or > 250 µg/ft^2^, depending on the location) and elevated soil lead (> 400 ppm).

Lead level/sociodemiographic variable	Unadjusted		Adjusted	
Elevated interior lead (*n* = 109)						
Income < $30,000		2.3	(1.0, 5.2)		2.8	(0.5, 15.9)
African-American race*a*		0.7	(0.3, 1.5)		0.8	(0.2, 3.9)
Elevated soil lead		21.2	(7.2, 62.7)		12.7	(2.9, 55.7)
Rent home		2.0	(0.8, 4.5)		0.5	(0.1, 2.3)
House located near busy street		0.7	(0.2, 3.0)		2.6	(0.3, 23.4)
House built pre-1946		12.8	(4.7, 35.1)		4.5	(0.8, 26.0)
Elevated soil lead (*n* = 90)						
Income < $30,000		1.2	(0.5, 3.0)		0.7	(0.2, 2.1)
African-American race*a*		0.7	(0.3, 5.7)		0.7	(0.2, 2.2)
Rent home		2.3	(0.9, 5.7)		2.7	(0.9, 7.9)
House located near busy street		0.4	(0.1, 2.3)		3.2	(0.5, 21.3)
House built pre-1946		82.0	(10.3, 651.5)		8.9	(3.1, 26.0)
**a**Reference group is white race.						

Soil data were geocoded to the 55 census tracts for which soil data were available for both pre- and post-Hurricane Katrina time periods. We compared the soil lead levels and assessed the difference in samples between the 2000 and the 2007–2008 surveys. In the 2000 survey, soil lead ranged from 25 to 1,789 ppm; in the 2007–2008 survey the range was 10–24,000 ppm. The median soil lead level from the 2000 survey was 408.1 ppm (interquartile range = 442.0 ppm), and the median lead level in the 2007–2008 survey was 560.0 ppm (interquartile range = 1,324 ppm), a 37% increase relative to the prestorm median. Overall, median levels for individual census tracts were significantly higher in the samples collected after Hurricane Katrina than in samples collected in 2000 (Wilcoxon signed rank test, *p* = 0.002).

## Discussion

The traditional lead distribution in New Orleans pre-Katrina was like that of many other older cities, with a higher concentration in and around deteriorating older housing. Besides age of housing, sociodemographic variables, including African-American race, low income, poverty, and renter status, have consistently been shown to be associated with elevated lead in urban cities (Gaitens et al. 2009; Jacobs et al. 2002; Lanphear et al. 2005). Hurricane Katrina disproportionately affected low-income, minority residents. The diaspora lacked the means to quickly return to the city, and our study sample reflects this repopulation pattern. Study households included a large proportion of nonminority homeowners living in areas of the city with low poverty. This demographic group would normally be considered to have a low risk for lead exposure; therefore, the widespread lead contamination in and around study homes—particularly the degree of soil contamination—was unexpected.

More than 60% of households had an elevated lead sample either inside or outside of the home. Nearly half the homes with bare soil had elevated soil lead, and 27% of those homes had soil lead > 1,200 ppm, three times the HUD/U.S. EPA standard. Neither soil nor interior dust lead levels were significantly associated with sociodemographic variables commonly found to be related to elevated lead, although low income was nonsignificantly associated with interior lead in adjusted models. We hypothesize that lead contamination may be the result of the unprecedented amount of home renovation and demolition that was required as a result of Hurricane Katrina damage both in high- and low-income neighborhoods.

In New Orleans, approximately 135,000 structures sustained hurricane damage, with major or severe damage to 105,000 of these homes. As of May 2009, an estimated 9,000 homes had been demolished and thousands more repaired or renovated (Brookings Institute 2009; HUD 2006). Both the renovation and demolition of these structures, many of which were built before 1950, pose a potential new lead exposure source and a potential health hazard for children (Chou et al. 2010; U.S. EPA 2006). Numerous studies have shown that when lead-safe practices are not employed during renovation, lead is dispersed into the environment and residential soil becomes contaminated (Clark et al. 2004; Dixon et al. 2007; Lanphear and Roghmann 1997; Reissman et al. 2002). Demolition activity is also a source of environmental lead and has been associated with an increase in children’s blood lead levels (Farfel et al. 2003, 2005; Rabito et al. 2007).

We found that the median soil lead level for the sampled homes was 37% higher than the median soil lead level for samples collected for a 1998–2000 lead survey. Our finding that soil lead levels in samples collected from the same census tracts in 2007–2008 were higher than prestorm levels contradict those from two soil lead distribution studies conducted after Hurricane Katrina (Walsh et al. 2006; Zahran et al. 2010). The U.S. EPA Louisiana Department of Environmental Quality survey found no change in the distribution of soil lead levels, and Zahran et al. (2010) reported a 46% decrease in median soil lead from pre-Katrina levels. Both of these studies were conducted in the immediate aftermath of Hurricane Katrina and before most reconstruction activity. Furthermore, the reduction in soil lead observed immediately after the storm may have reflected relatively low levels in soil sediment from surrounding water bodies, which was deposited during widespread flooding of the city (Zahran et al. 2010). Natural and anthropogenic activities can redistribute soil lead, and it was predicted by Potera (2010) that the cleaner soil sediment brought by Hurricane Katrina may not persist. The ATSDR (2007) specifically warned of the risk of lead dispersion as a result of home renovation and demolition and predicted that if lead-safe practices were not employed, the level and the extent of soil and interior contamination would increase.

As a result of Hurricane Katrina’s widespread destruction, homes previously in good condition required renovation, and the subsequent disturbance of lead in old homes makes the risk of lead exposure universal. In this population, 64.2% of the homes were built before 1946, a period when lead paint was in widespread use. Lead paint can be released directly into the soil via air resuspension during renovation activities. Power sanding, a popular method of exterior paint removal, can release a large amount of lead dust into the environment. HUD (1995) estimated that 1 ft^2^ of pulverized lead paint produces a settled dust lead level of 9,300 µg/ft^2^. Although power sanding is prohibited in New Orleans by city ordinance (New Orleans, Louisiana, Code of Ordinances 2001), lack of oversight in the postdisaster environment resulted in widespread sanding of homes undergoing renovation. Therefore, it seems plausible that subsequent soil contamination may have occurred. If renovation/demolition activities are contributing to the high lead levels, the lack of association with commonly known sociodemographic factors is not surprising given the extent of housing damage.

There is abundant evidence that soil lead exposure is a significant contributor to blood lead levels in children (Clark et al. 2004; Dixon et al. 2007; Lanphear et al. 2005; Ren et al. 2006). Estimates of the contribution of exterior soil to indoor dust range from 20–30% (Culbard et al. 1988; Davies et al. 1985; Rutz et al. 1997) to as high as 85% (Fergusson and Kim 1991; Roberts et al. 1991; Trowbridge and Burmaster 1997). Urban soils can integrate lead from numerous sources, including paint on the exterior of homes, leaded gasoline emissions, and incinerator or industrial lead emissions that have accumulated in the environment (Mielke 1999; Mielke et al. 2005). Because regulations are in place to limit industrial emissions and because leaded gasoline and paint have been banned, of particular concern is new contamination as a result of renovation and demolition of old structures. According to a 1998–2000 national survey (Jacobs et al. 2002), 24 million housing units contain lead-based paint hazards; these housing units serve as a reservoir of lead hazards that can pose a risk to children via dust and soil for years to come. The preponderance of old homes coupled with extensive renovation/demolition activities and high dust and soil lead suggests that children in New Orleans are at substantial risk of environmental lead exposure in and around their homes. An alternate explanation is that soils contaminated with heavy metals may have been carried by flood water and redeposited to new locations post-Katrina (Presley et al. 2010). However, regardless of the actual source of contamination and the findings from the pre- and post-Katrina comparative analysis, our findings indicate that lead contamination—particularly soil contamination—is prevalent and has significant public health implications for residents, especially children. The current targeted screening and public health intervention efforts to prevent childhood lead poisoning may need to be expanded to capture a population that previously was not considered at risk of environmental lead exposure.

Regulatory action is an effective tool for reducing lead exposure. The key, however, is the extent to which regulations are enforced and their degree of coverage. A U.S. EPA rule governing renovation on child-occupied structures built before 1978 was adopted in April 2008 (U.S. EPA 2008b). Although the rule provides a formal framework for lead-safe work practices, it has several limitations. First, it includes work performed by contractors and exempts work performed by homeowners, tenants, and day laborers. In a study of home renovation among children with lead levels ≥ 20 µg/dL in New York, 14% of elevated levels were attributed to renovation, and owners or tenants performed 66% of that work (CDC 2009). The rule also exempts homes without children. Although a child may not currently reside in the home, there can be no prediction for future occupancy. Furthermore, children residing near homes where lead dust is dispersed are put at risk from resuspension of lead, particularly in high-density areas, such as urban cores, where the vast amount of old housing exists. Testing urban soils should be a priority, and policy officials should support efforts to halt power sanding and other work practices that result in resuspension of lead into the environment.

This study has several limitations. Although households were selected by stratified random sample, the overall response rate was low at 32.5%. Although likely an underestimation because of the conservative approach taken to define occupancy, there were no reliable population estimates because occupancy was a dynamic and fluid process post-Katrina. The possibility of selection bias due to differential participation by households concerned about lead hazards should be considered; however, the primary goal of the project was measurement of allergens and mold in homes, and mold exposure was the principal health concerns for residents returning to New Orleans after the storm. Furthermore, media extensively reported the U.S. EPA finding of no new lead exposure in the wake of the storm; therefore, we feel it is unlikely that participation was motivated by concern for lead hazards. A further limitation of the study is the lack of information on renovation activities for participating households and structures nearby to support the ad hoc hypothesis that renovation activities are contributing to the high residential lead levels. Although > 86% of households in this study reported that they had completed renovations, were in the process of renovating, or still needed renovation, no data were collected as to the extent of damage or what type of renovations had taken place; therefore, renovation status could not be included in the multivariate model, and the role that renovation and demolition may have played remains unknown. Finally, our comparison of soil lead data pre- and post-Katrina has some limitations. Although our approach was similar to that of other studies, it is likely that differences in sampling methodology may explain some of the observed differences in census-tract–specific soil lead levels. In the present study, samples were collected exclusively from soil around participating houses. In the 2000 soil survey (Mielke et al. 2000), although data were collected primarily from around the house, some samples were collected near streets in residential neighborhoods. Moreover, the number of soil samples taken per census tract varied between the surveys.

## Conclusion

To our knowledge, this is the first study of residential lead hazards in New Orleans in the aftermath of Hurricane Katrina. Survey results indicate that 61% of homes had lead levels that exceed the U.S. EPA standards, independent of race, income, and ownership status. New Orleans children are at risk for elevated blood lead levels, including children who were not considered at high risk previously and for whom lead reduction has been considered a public health success. Enhanced surveillance and lead hazard mitigation efforts are needed to safeguard the health of New Orleans residents.
